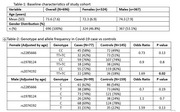# Human *ACE2* Polymorphisms and Susceptibility to COVID‐19 Infection in the TLSA Cohort

**DOI:** 10.1002/alz70860_106504

**Published:** 2025-12-23

**Authors:** Prathima Arvind, Piu A Das, Sohan Angelo A, Abhishek Mensegere Lingegodwa, Krithika Subramaniam, Khader Valli Rupanagudi, Albert Stezin, Amitha A CM, Gauri Mullerpattan, Vidhya R, Jonas S Sundarakumar, Thomas Gregor Issac

**Affiliations:** ^1^ Centre for Brain Research, Indian Institute of Science, Bangalore, Karnataka, India; ^2^ Center for Brain Research, Bangalore Urban, Karnataka, India; ^3^ Centre for Brain Research, Indian Institute of Science, Bangalore, India; ^4^ Center for Brain Research, Bangalore, India; ^5^ Center for Brain Research, Bangalore Urban, India

## Abstract

**Background:**

Studies on host‐pathogen interactions have identified human *ACE2* as a key receptor on host cells that facilitates infection by the coronavirus (COVID‐19). Further research has highlighted significant differences in the allele frequencies of single nucleotide polymorphisms (SNPs) within the *ACE2* gene, which contribute to varying levels of susceptibility to COVID‐19 in European and Asian populations (1, 2). This study aims to explore the association between *ACE2* gene polymorphisms and COVID‐19 infection within the TATA Longitudinal Aging Study (TLSA) cohort.

**Method:**

The study utilized baseline data from the TLSA cohort (*N* = 696). Quality control (QC) and phenotype‐genotype analyses were conducted using PLINK v1.9.0. Following QC procedures, a total of 480 participants were included in the genotype‐phenotype analysis.

**Result:**

The cohort consists of 696 participants with genetic data, having a mean age of 73.6 years (±7.6 years) (Table 1). 46% of the participants are female, while the remaining are male. Our study investigated the association between *ACE2* gene polymorphisms (rs2285666, rs1978124, and rs2074192) and COVID‐19 susceptibility in the TLSA cohort, with stratification by sex and adjustment for age. In an additive model analysis, rs2074192 (TT+TC) genotype was significantly associated with an increased risk of COVID‐19 infection in females with an odds ratio (OR) of 1.69 (95% CI: 1.06‐2.68, *p* = 0.02), suggesting a potential genetic predisposition. However, rs2285666 with an OR of 0.73 (95% CI: 0.58‐1.44, *p* = 0.13) and rs1978124 with an OR of 0.9 (95% CI:0.49‐1.09, *p* = 0.8) showed no significant associations. In males, none of the tested polymorphisms, including rs2074192 with OR of 1.59, (95%CI:0.90‐2.83, *p* = 0.13) (Table 2).

**Conclusion:**

Our findings indicate a possible sex‐specific genetic influence of *ACE2* polymorphisms on COVID‐19 susceptibility, with rs2074192 showing a significant association in females but not in males. These results highlight the need for further research to explore the biological mechanisms underlying this association and its implications for personalized risk assessment and targeted interventions in COVID‐19.